# Rubella virus mutations that confer resistance to inactivation at low pH

**DOI:** 10.1128/jvi.00255-25

**Published:** 2025-05-16

**Authors:** Pratyush Kumar Das, Margaret Kielian

**Affiliations:** 1 Department of Cell Biology, Albert Einstein College of Medicine 2006, Bronx, New York, USA; St. Jude Children's Research Hospital, Memphis, Tennessee, USA

**Keywords:** rubella virus, rubivirus, virus fusion, virus entry

## LETTER

**Fig 1:**
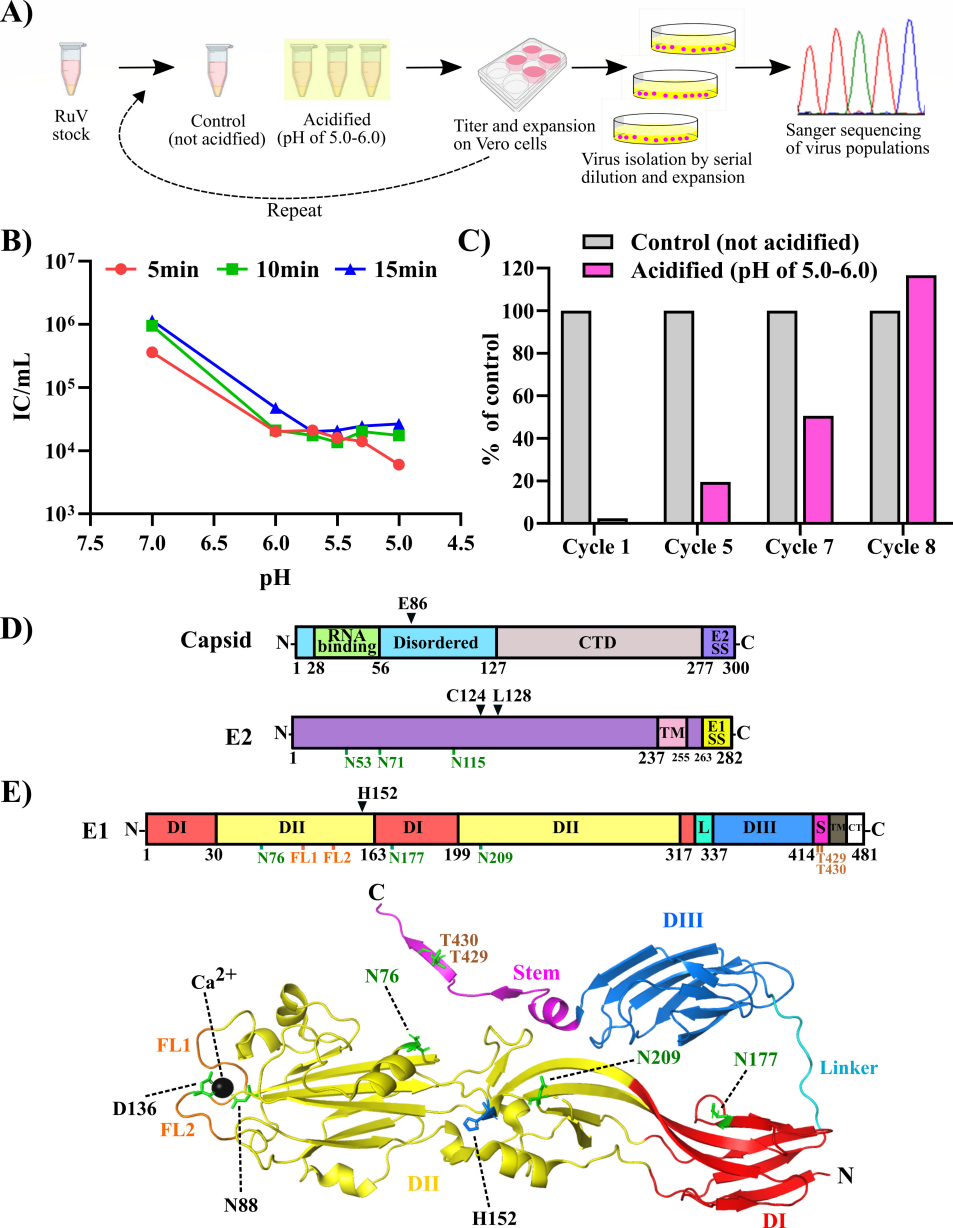
Selection of low pH-resistant RuV mutants. (**A**) Schematic of the selection process. Independent aliquots of RuV stock were treated for 10 min at 37°C at a pH between 5 and 6, adjusted to neutral pH, and expanded by growth on Vero cells. Selection and expansion cycles were repeated eight times, and viruses were isolated and sequenced. (**B**) Sensitivity of parental RuV to treatment at 37°C for the indicated pH and times. (**C**) Example of enrichment of low pH-resistant RuV. Low pH inactivation of lineage 2 virus is shown after the indicated selections (cycle 1, pH 6; cycle 2, pH 5; cycle 7, pH 5.7; cycle 8, pH 5.3). Virus titers after each treatment are shown as the percentage of a parallel pH 7 control. (**D and E**) Positions of the structural protein residues assessed for effects on RuV acid resistance. Protein domain boundaries and features are numbered at the bottom of the linear diagrams, with the positions of the residues tested by mutagenesis (capsid E86, E2 C124, E2 L128, and E1 H152) indicated by black text and an inverted black triangle above the linear diagrams. (**D**) Top panel: Diagram of capsid showing the disordered region (cyan), including the RNA-binding domain (green), the C-terminal domain (CTD, latte), and the E2 signal sequence (E2SS, violet). Bottom panel: E2 diagram showing the transmembrane domain (TM, flamingo) and E1 signal sequence (E1SS, titanium), with the sites of N-linked glycosylation labeled in green (2). (**E**) Top panel: Linear E1 diagram showing domains DI (red), DII (yellow), DIII (blue), linker (L, cyan), stem (S, magenta), transmembrane (TM, dark gray), and cytoplasmic tail (CT). Fusion loop (FL) 1 (aa 88 to 93) and FL 2 (aa 131 to 137) are labeled in orange, and N- and O-linked glycosylation positions are labeled with green and sepia, respectively (2). Bottom panel: The post-fusion RuV E1 structure (PDB: 4ADJ) (6), color-coded as in the upper panel and indicating the associated Ca^2+^ ion (black sphere), the Ca^2+^ ion-coordinating residues N88 of FL1 and D136 of FL2, the N-linked (green) and O-linked (sepia) glycosylation sites, and H152 (blue).

Rubella virus (RuV) is an enveloped positive-strand RNA virus that can cause miscarriage or severe congenital birth defects in humans (1). RuV infects cells by endocytic uptake and a Ca^2+^ and low pH-dependent membrane fusion reaction that occurs in early endosomes (1–5). While the requirement for Ca^2+^ and its interaction with the RuV fusion protein E1 have been characterized (3, 5, 6), the regulation of RuV fusion by low pH is poorly understood. Our previous studies showed that RuV membrane fusion is optimal at ~pH 6.2 (3). Similarly, RuV infectivity is decreased by treatment at ~pH 6.2 or below in the absence of target membranes, a treatment that converts E1 to the post-fusion conformation (3). Here, we developed a selection strategy to identify residues that confer RuV resistance to low pH (Fig. 1A). Treatment at pH 6.0 or below for 5–15 min reduced the titer of the M33 strain of RuV by ~2 logs (Fig. 1B). RuV was subjected to repeated cycles of low pH treatment and expansion on Vero cells until low pH resistance of the independent virus stocks was observed (Fig. 1A and C). Viruses were then isolated by serial dilution, and mutations in their structural proteins, capsid (Cp), E2, and E1, were identified by Sanger sequencing (Table 1 and Fig. 1D and E). All of the isolates contained the mutation E1 H152N, along with either E2 C124R or [E2 L128P + Cp E86Q].

**TABLE 1:** Mutations present in RuV isolates after selection for low pH resistance

Independent isolate	Capsid^*a*^	E2^*a*^	E1^*a*^
1	P149**T** (CCC to **ACC**)	S104**P** (TCT to **CCT**) C124**R** (TGC to **CGC**)	H152**N** (CAC to **AAC**)
2		C124**R** (TGC to **CGC**)	H152**N** (CAC to **AAC**)
3	P80P^*b*^ (CCC to CCT) E86**Q** (GAA to **CAA**)	L128**P** (CTC to **CCC**) G204G^*b*^ (GGT to GGC)	H152**N** (CAC to **AAC**)
4		C124**R** (TGC to **CGC**)	H152**N** (CAC to **AAC**)
5	E86**Q** (GAA to **CAA**)	L128**P** (CTC to **CCC**)	H152**N** (CAC to **AAC**)
6	L177L^*b*^ (CTG to CTA)	C124**R** (TGC to **CGC**)	H152**N** (CAC to **AAC**)

^*a*^ Position of the amino acid change is indicated with acid-selected residue in bold and corresponding codon in bold in parentheses.

^*b*^ Synonymous change.

**Fig 2:**
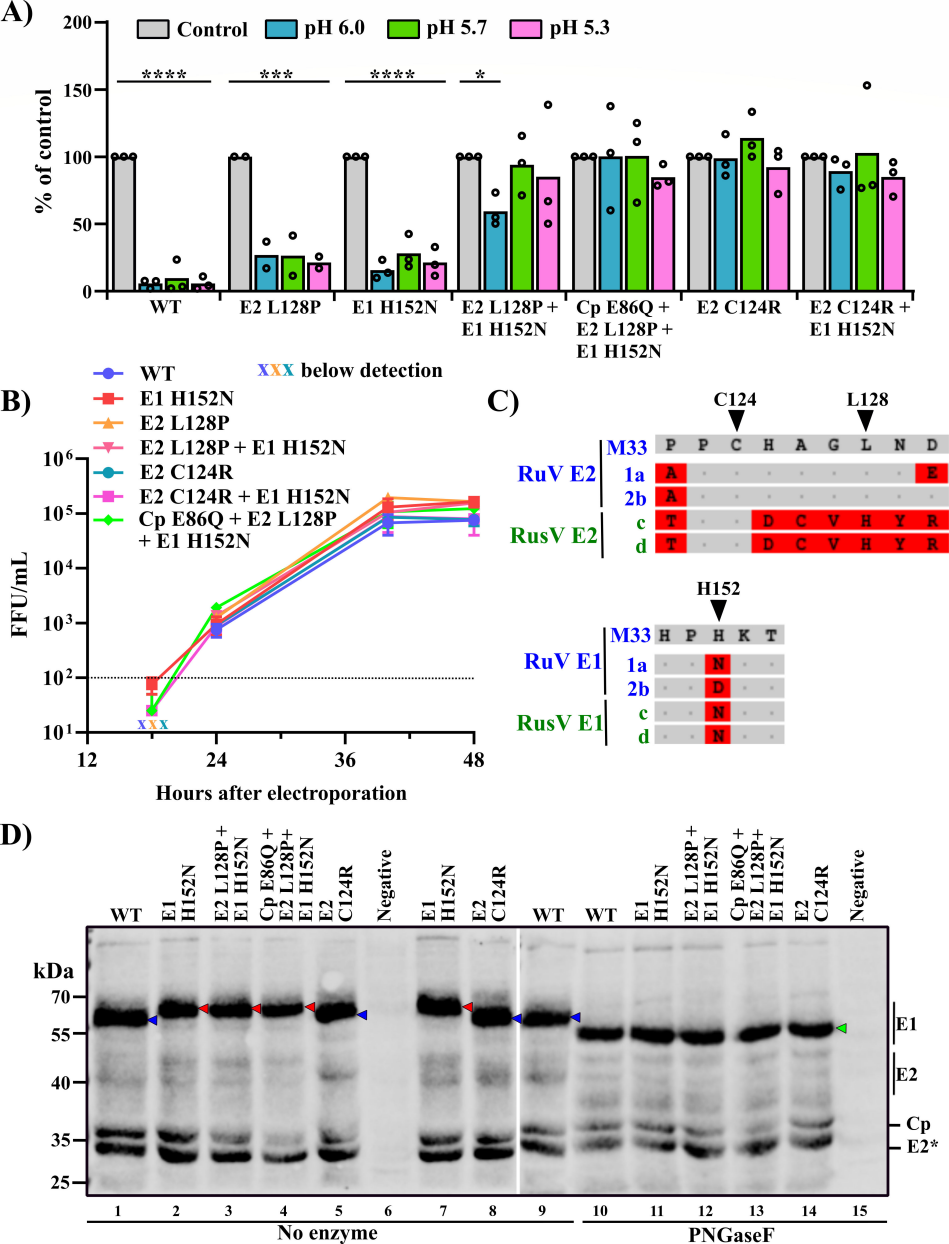
Characterization of low pH-resistant RuV mutants. (**A**) Low pH sensitivity of engineered RuV mutants. Aliquots of WT or mutant P1 virus stocks (diluted to ~10^5^ FFU/mL) were incubated at the indicated pH (or pH 7 as a control) for 10 min at 37°C and titered. Statistical analyses were carried out by two-way ANOVA with Dunnett’s multiple comparisons tests against the control sample. ****P < 0.0001; ***P < 0.001; *P < 0.05. (**B**) Growth kinetics of low pH-resistant RuV mutants. *In vitro* transcribed RNAs from WT or mutant infectious clones were electroporated into BHK cells. Supernatants were collected at the indicated time points and titered (mean of *n* = 2 with range). The limit of virus detection is shown as a dotted line. The data in A and B are from two to three biological experiments using two independent infectious clones of the indicated mutant. (**C**) Sequence comparison of key residues involved in RuV acid resistance. Structural protein ORFs from RuV M33 (M33) [AEN94516.1] (11), RuV genotype 1a (1a) [QBZ96524.1], RuV genotype 2B (2b) [WBQ85901.1], and RusV isolates from capybara (**C**) [QKO01651.2] and donkey (**D**) [QKO01649.2] (8) were aligned using the NCBI Virus website. Regions encompassing E2 C124 and L128 and E1 H152 are shown in the upper and lower panels, respectively. Non-conserved residues are highlighted in red. Note that the E1 sequence ^152^HKT^154^ in RuV M33 is ^152^NKT^154^ in RuV 1a and RusV, generating an additional N-linked glycosylation site in those viruses (9). (**D**) N-linked glycosylation analysis of acid-resistant mutants. Lysates of Vero cells infected for 72 h with WT or mutant RuVs were treated with no enzyme (lane 1–9) or PNGaseF (lane 10–15) and analyzed by Western blotting with RuV polyclonal antibody. White space between lanes 8 and 9 indicates where two blot images from the same experiment have been combined. In the no enzyme samples, the E1 proteins that co-migrate with WT E1 are indicated by a blue arrowhead, while the slower migrating E1 in the mutants containing E1 H152N is indicated by red arrowheads. The PNGaseF-sensitive E1 bands are indicated by a green arrowhead at the right of the gel. E2* indicates a probable non-glycosylated form of E2. Images are representative of three independent experiments.

The role of these residues in low pH resistance was tested by engineering them into the pBRM33 RuV infectious clone (7). Low pH treatment of the resultant virus stocks showed that either the E2 L128P or the E1 H152N mutation caused a small increase in low pH resistance, while the double mutant [E2 L128P + E1 H152N] and the single mutant E2 C124R were highly resistant to low pH inactivation (Fig. 2A). Resistance was marginally improved by the addition of Cp E86Q to the double mutant, while the addition of E1 H152N to E2 C124R did not detectably affect virus resistance. The WT and mutant RuVs showed comparable growth kinetics in BHK cells (Fig. 2B). We aligned the sequences surrounding E2 C124, E2 L128, and E1 H152 from RuVs and Rustrela virus (RusV), a related rubivirus that causes severe neurological symptoms in a range of mammalian species (8) (Fig. 2C). E2 C124 is conserved between the two rubiviruses, while E2 L128 is not. RuV M33 E1 H152, which was substituted by N in all of our mutant isolates, is also N in RuV genotype 1a and in all of the reported RusV isolates and is D in RuV genotype 2b. The E1 H152N mutation is predicted to produce a new N-linked glycosylation site (9). Comparison of the WT and mutant proteins showed that the E1 proteins from all of the mutants that contained E1 H152N had a slower migration in SDS-PAGE, which was lost when the proteins were digested with PNGaseF to remove N-linked carbohydrates (Fig. 2D). Our results thus indicate that the E1 H152N mutation does confer an additional glycosylation site, but that this alone does not promote strong low pH resistance. This is consistent with our finding that RuV produced in CHO cell lines with altered or absent N-linked glycosylation (10) does not differ in acid sensitivity (Mathieu Dubé, unpublished results).

Our data show that the E2 C124R mutation promoted low pH resistance. This mutation is perhaps surprising, as the high conservation and even number of C residues in the RuV E2 ectodomain suggest that the cysteines may all be paired in intramolecular disulfide bonds. The [E2 L128P + E1 H152N] double mutant was also very resistant to low pH, suggesting that these two mutations could act together to stabilize the E2E1 prefusion heterodimer. The prefusion structures of E1 and of the E2E1 dimer remain unresolved, but analysis from cryo-EM tomography studies suggests that E2 may be located at the base of E1, perhaps allowing an interaction of E2 L128P and E1 H152N to stabilize the E1 prefusion structure (12). We recently reported that a W448R mutation in the E1 transmembrane domain decreased the pH threshold of RuV membrane fusion from ~pH 6.2 to pH 5.5 (13), and thus, other regions of E1 can be involved in regulating pH sensitivity. Studies of the alphavirus Semliki Forest virus (SFV) show that WT SFV fuses at pH ~6.2 in early endosomes, while a low pH-resistant SFV mutant fuses at pH ~5.5 in late endosomes (14–16). The low pH resistance of the RuV mutants identified in this study suggests that they would similarly fuse in late endosomes.

RuV E1 is a class II membrane fusion protein (6). Studies of pH regulation of alphavirus class II fusion proteins indicate that both the dissociation of the E2-E1 heterodimer and independent conformational changes in E1 affect the pH dependence of alphavirus fusion and low pH inactivation (17, 18). It is unclear at present how the mutations we have identified regulate the pH dependence of RuV, but both heterodimer and E1 conformational changes may play a role.

### Cell lines and antibody

BHK-21/WI-2 and Vero cells were cultured at 37°C with 5% CO_2_ in Dulbecco’s modified Eagle’s medium (DMEM) containing 25 mM D-glucose, 4 mM L-glutamine, 100 U penicillin/mL, and 100 µg streptomycin/mL, supplemented with 10% FBS (Vero) or 5% FBS plus 10% tryptose phosphate broth (BHK-21). Goat polyclonal antibody to RuV (pAb) was obtained from MilliporeSigma (Catalog No. AB1060, Burlington, MA).

### Viruses

Primary (P0) stocks of M33 WT RuV or mutant RuVs were generated from the pBRM33 WT RuV infectious clone (a gift from Dr. Tom Hobman) (7), or the mutant clones produced as described below. Viral RNAs were transcribed using SP6 polymerase and electroporated into BHK-21 cells (19). Supernatants were collected at 40 h post-electroporation and titered, and working stocks (P1) were generated by passage on Vero cells for 72 h.

### Virus titration

Virus samples were titered either by the infectious center assay (ICA) or focus formation assay (FFA) on Vero cells, as described (19). In brief, 96-well plates were seeded with 12,000 Vero cells/well 24 h before infection and then inoculated with tenfold serial dilutions of virus for 4 h. The media were then replaced with DMEM + 5% FBS containing either 20 mM NH_4_Cl (ICA) or 2% carboxymethylcellulose (FFA). At 48 h post-infection, cells were fixed with 4% paraformaldehyde, stained with RuV pAb, and visualized by appropriate fluorescently tagged (ICA) or peroxidase-labeled (FFA) secondary antibody. The ICA was scored by fluorescence microscopy, while the FFA was quantitated using an Immunospot S6 CTL analyzer.

### pH treatment and selection of acid-resistant RuV mutants

pH treatments used virus diluted into pH medium [RPMI 1640 without sodium bicarbonate plus 10 mM HEPES pH 7.0, 10 mM MES pH 7.0, 0.2% BSA]. Pre-titrated volumes of 0.5 N acetic acid were added to obtain the indicated pH, and samples were incubated at 37°C for 5–15 min, then adjusted to neutral pH by the addition of 1 M HEPES (pH 8). For mutant selection, aliquots of the RuV P1 stock were incubated at a pH range from 6.0 to 5.0 for 5–10 min at 37°C, titered, and expanded by passage on Vero cells at low MOI. Low pH resistance was observed after eight cycles, and viruses were isolated by serial dilution on 96-well plates and expanded on Vero cells (19).

### Sequencing of RuV structural region

Sequencing of the RuV structural ORF (19) was performed using RNA extracted from ~10^6^ IC of each virus isolate (~10^6^ IC/mL) using the RNeasy Mini Kit (Qiagen, Hilden, Germany) and reverse transcribed using ProtoScript II First Strand cDNA Synthesis Kit (NEB, Ipswich, MA) and the RuV-specific primer (5′CGAATTCAAGCTTTTTTTTTTTTTTTTT3′). The *HindIII* restriction enzyme site used for linearization of the pBRM33 RuV infectious clone for *in vitro* transcription is underlined. The RuV structural ORF was PCR-amplified using the Q5 high-fidelity polymerase (NEB, Ipswich, MA) with high GC enhancer supplement and appropriate overlapping primer pairs and analyzed by Sanger sequencing (Genewiz, South Plainfield, NJ) (19).

### Cloning and mutagenesis

Selected mutations in the structural ORF were inserted individually or in various combinations into the RuV infectious clone. The E1 H152N, E2 L128P, and E2 L128*P* + E1 H152 mutants were obtained by ligating mutagenized PCR fragments, individually or in combination, into pBRM33 to produce pBRM33-E1H152N, pBRM33-E2L128P, and pBRM33-E2L128P-E1H152N. To insert the Cp E86Q or E2 C124R mutations, the corresponding residues were first changed in an intermediate pUC19_XbaI_SbFI_M33 plasmid by standard PCR-based mutagenesis (20), and the mutagenized fragments were then ligated into the appropriate pBRM33 WT or mutant infectious clone. Two independent clones of each mutant (one for pBRM33-E2L128P) were produced and confirmed by Sanger sequencing.

### Virus growth assay

Ten microliters of each *in vitro* transcription reaction was electroporated into BHK-21 cells (19). Cells were cultured in 35-mm plates, and supernatants were harvested at indicated time points and titered by FFA.

### Glycosylation assay

Vero cells in 35-mm plates were inoculated with virus P1 stocks at MOI 0.1 FFU/cell. At 72 h post-infection, cells were washed three times with cold PBS and lysed in buffer containing 50 mM Tris pH 7.4, 100 mM NaCl, 1 mM EDTA, 1% Triton X-100, 2 µg aprotinin/mL, and 1 mM PMSF. Samples were clarified by centrifugation, denatured in the presence of SDS and DTT at 100°C for 10 min, and digested with PNGaseF for 90 min at 37°C (19), according to the manufacturer’s instructions (NEB, Ipswich, MA). Samples were then adjusted to 0.5% SDS and 3 mM DTT, heated at 70°C for 1 min, and analyzed by SDS-PAGE and Western blotting with RuV pAb.
